# Supplementary material for: Distinct solvation patterns of OH− versus H3O+ charge defects at electrified gold/water interfaces govern their properties
Source: Nat Commun. 2025 Sep 19;16:8325. doi: 10.1038/s41467-025-63832-1 (PMC12449469; doi:10.1038/s41467-025-63832-1)
Supplement: Supplementary file 3 — LaTeX Supplementary File [file 41467_2025_63832_MOESM3_ESM.pdf]

Supplementary Information: Distinct Solvation patterns of OH^- versus H_3O^+ charge defects at electrified gold/water interfaces govern their properties

Chanbum Park,[1*] Soumya Ghosh,[1,2] Harald Forbert[3] and Dominik Marx[1]

[1]Lehrstuhl für Theoretische Chemie, Ruhr-Universität Bochum, 44780 Bochum, Germany
[2]Present address: Tata Institute of Fundamental Research Hyderabad, Hyderabad, Telangana 500046, India
[3]Center for Solvation Science ZEMOS, Ruhr-Universität Bochum, 44780 Bochum, Germany
chanbum.park@theochem.ruhr-uni-bochum.de

Contents

**Supplementary Note 1**	**Potentiostat**	**2**
**Supplementary Note 2**	**Collective variables to define the charge defects**	**4**
**Supplementary Note 3**	**Radial distribution functions in a slab geometry**	**5**
**Supplementary Note 4**	**Trajectories of H_3O^+**	**6**
**Supplementary Note 5**	**Orientation and hydrogen bonding of water**	**8**
**Supplementary Note 6**	**Orientation and density profiles of H_3O^+**	**10**
**Supplementary Note 7**	**Trajectories of OH^-**	**12**
**Supplementary Note 8**	**Density and excess charge profiles of H_3O^+ versus OH^-**	**13**
**Supplementary Note 9**	**Net atomic charges**	**17**

Supplementary Note 1 Potentiostat

To generate finite surface charge densities at metal/water interfaces during ab initio molecular dynamics (AIMD) [1] simulations in a continuously controllable manner, we employ a supercell method,[2] where the computational voltage is obtained from the dipole correction [3] at the center of the vacuum region between a computational counter electrode (Ne in this case) and the bottom-most layer of the electrode of interest (here Au). In the present investigation, we use this approach to disclose the possible impact of finite potential bias on the structural and dynamical properties of two charge defects, namely the hydronium (H_3O^+) and hydroxide (OH^-) ions, at the electrified interface of pure water with a perfect gold surface. In order to being able to properly focus on the solvation and charge transfer properties of these two *generic* charge defects, we do not add electrolyte salts to our acidic and alkaline aqueous solutions. As a consequence of our choice, no electric double layer can form which would be fundamentally necessary to compute the proper dependence of the surface charge density on the applied electrode potential. In addition, the absence of a double layer at the interface results in incomplete screening of the electrostatic field from the Au surface. The *computational* voltage, which is calculated using the dipole correction [3] within the present approach [2], does not correspond to the voltage drop across the double layer as measured in typical electrochemical experiments. Nevertheless, this *computational* voltage can still serve as a measure of the applied bias in the system: As we demonstrate below in detail, the surface charge density can be controlled systematically and continuously by changing the *computational* voltage although the quantitative relation of applied voltage and surface charge density cannot be accessed, leaving comparisons to experiments at the qualitative level; see below for a detailed discussion of the caveats of the present approach as used here and their implications. Despite these clear limitations, the current AIMD-based setup does allow us to access the changes of solvation structures and charge transfer reactions underlying the structural diffusion of H_3O^+ and OH^- at these electrified interfaces with respect to their well-known structures and distinct Grotthuss diffusion mechanisms in pure bulk water [4] that serves as our reference system.

In the method we use,[2] the effective nuclear charge of the Ne atoms that establish the computational counter electrode is modified to charge the metal electrode of interest; we refer the interested reader to Ref. [2] for background, validation and details of this method. By increasing (or decreasing) the effective nuclear charge of each Ne atom from Z_{Ne} to $Z_{\text{Ne}} + \delta Q/n$, where δQ and n are the net excess charge and the total number of Ne atoms, respectively, additional electrons (or electron holes) are created at the conduction band (or valence band) of the Ne atoms to conserve the net charge; note that δQ does not need to be integer and thus allows one to continuously change the computational bias. The additional electrons in the conduction band are redistributed to the Au electrode during electronic structure calculations since the conduction band minimum of the Ne is much higher than the Fermi level. For electron holes, electrons from the Au electrode are accumulated in the valence band of the Ne atoms.

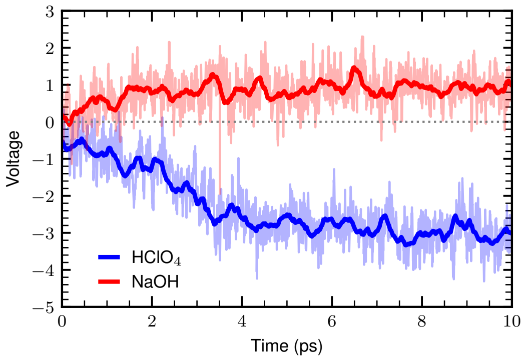

Supplementary Figure 1: Evolution of the instantaneous computational voltages (light colors) and the averaged computational voltages (thick lines) for the acidic ($HClO_4$) and alkaline (NaOH) systems as a function of AIMD sampling time.

In the actual implementation, instead of balancing the number of electrons on each species according to their modified effective nuclear charge, the fractional occupation number of the orbitals are modified according to the net excess charge, δQ. This net transfer of electrons, with reference to the isolated species, generates the surface charge δQ at the metal electrode. The charged electrode attracts or repels

water molecules and ions. The ensuing distributions of ions and oriented water molecules at the Au surface cause potential drops across the cell. The potential drop is compensated by a dipole correction in the middle of the vacuum region so that there is no net dipole across the simulation cell.[3] This allows one to compute the instantaneous computational voltage within the cell as well as the overall periodic variation of the potential.

To reach the target computational potential, the core charge of the Ne atoms is updated according to the target computational voltage and the current computational voltage. The additional charge δQ_{add} to maintain the target computational voltage is determined as[2] $\delta Q^{\text{add}} = \beta(V^{\text{target}} - V^{\text{aver}})$, where the value of β is chosen to be 1 eV^{-1} while the maximum possible charge update value is set to 0.01 e. In practice, the current (instantaneous) computational voltage is averaged over 200 fs of AIMD simulation, denoted as V^{aver}, and compared with the target computational voltage V^{target} to update δQ^{add} accordingly. Following the protocol validated in Ref. [2], a fraction of the snapshots were used to compute the achieved averaged computational potential V^{aver} as

$$V^{\text{aver}} = \sum_{i>\alpha N_0}^{N_0} V(i)/(N_0 - \alpha N_0), \tag{1}$$

where $V(i)$ is the current computational potential at snapshot i and N_0 is the number of steps for each run (200 fs). We set $\alpha = 0.8$ and the target computational potential drop is increased/decreased every 200 fs by $\pm$ 0.2 V. Supplementary Fig. 2 illustrates this approach and demonstrates that the instantaneous computational voltage (blue line) fluctuates in a stable manner with respect to the target computational voltage (horizontal yellow line), thus allowing one to compute after the relaxation and equilibration phases the surface charge density that corresponds to the computational bias potential within the limitations explained in the text.

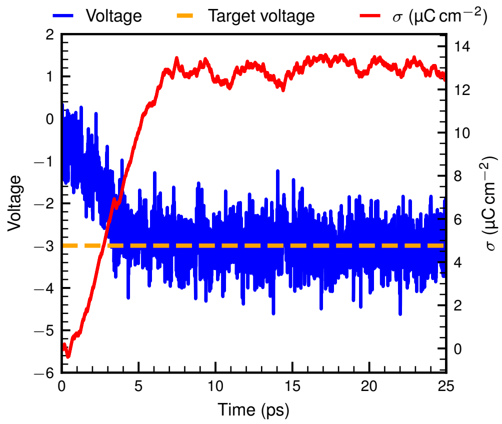

Supplementary Figure 2: Evolution of the instantaneous computational voltage (blue solid line) and the corresponding surface charge density (red solid line) for the acidic ($HClO_4$) system as a function of AIMD sampling time obtained by iteratively updating δQ^{add} to establish the desired target computational voltage $V^{\text{target}} = -3$ V following the approach described above in the text.

As a note of caution we stress the fundamental limitation that the approach as used here provides only a surface charge density corresponding to the *computational* bias potential since our setup does not include electrolyte ions. As already mentioned above, no electric double layer and the corresponding double-layer capacitance is established at our gold/water interface – as would be required if our aim was to model full electrochemical cells at work with a proper relation of electrode potential and surface charge density. Our present aim, however, is to setup an electrified interface between an explicit gold electrode model in contact with pure water at which we can investigate, depending on a continuously controllable surface charge density such as demonstrated in Supplementary Fig. 2, the *genuine* structural and dynamical properties of hydronium and hydroxide charge defects (but, unfortunately, without being able to properly connect that surface charge density to the applied bias potential). In particular, this includes the specific charge transfer reactions of these defects within interfacial water close to negatively and positively charged metal electrodes, which underlie their distinct *"pristine"* Grotthuss-style migration mechanisms in such *idealized* acidic and alkaline aqueous environments, respectively.

We point out that these insights are only accessible in the strict *absence* of any perturbing electrolyte ions, as offered by the present approach, since ions at aqueous surfaces are known to strongly alter the interfacial H-bond network due to the need to establish their own hydration shells, thus interfering in a concentration-dependent manner with those of the H_3O^+ and OH^- charge defects.

In our computational framework, the voltages are referenced to the potential of zero charge (pzc) condition of the corresponding systems. It has been established that pzc-referenced voltages are sufficient to observe bias effects on the liquid structure near metal surfaces.[5, 6] Meanwhile, the pzc values for the present acidic and alkaline systems are −0.4 and −0.1 V, respectively. The computational voltages reported in the main text for these systems are referenced to the corresponding pzc values. For the acidic system, the mean voltage of the trajectories is approximately −2.9 V, while for the alkaline system, the voltages range from 0.7–1.0 V. Thus, we refer in the main text to bias potentials of −3 and 1 V for the acidic and alkaline systems, respectively. The additional charge on the Au slab, accumulated there by manipulating the effective nuclear charge of the Ne atoms within the present approach,[2] can be translated into a surface charge density (see Supplementary Fig. 2 for an illustration corresponding to the −3 V case) as summarized in Supplementary Table 1.

Supplementary Table 1: Relation of computational voltages and surface charge densities of the investigated systems as explained in the text; numbers in parentheses are estimated standard deviations.

Computational potential (V)	Surface charge density ($\mu C\,cm^{-2}$)
−3	−13(1)
−2	−9(1)
pzc	0
+1	+4(1)

Based on the data in Supplementary Table 1, we can correlate the surface charge densities obtained from our AIMD simulations with experimental values reported in the literature [7, 8] while keeping the caveat in mind that our present setup does not model the electric double layer and thus does not provide access to the electrochemical capacitance. Comparison to these experimental charge densities as a function of the electrode potential [7, 8] qualitatively demonstrates that our computational voltages as defined above are in the regime that should not induce any electrochemical reaction in the acidic and alkaline cases. This is important since such reactions would strongly interfere with the intrinsic molecular properties of the H_3O^+ and OH^- charge defects close to the gold electrode on which we focus in this investigation. For instance in case our acidic solutions, our most negative computational potential translates to a surface charge density of roughly $\sigma \approx -13(1)$ $\mu C\,cm^{-2}$ according to Supplementary Fig. 2 within the limitations of our setup as explained above. Although we cannot compare our computational bias potentials to experimental voltages for reasons explained above, we can qualitatively correlate the computed surface charge densities to corresponding experiments at low concentrations.[7] Our most negative computational bias potential for the simulated acidic $HClO_4$ solution corresponds to a surface charge density of about −13(1) $\mu C\,cm^{-2}$ according to the Supplementary Table 1. This is qualitatively consistent with experimental surface charge density versus electrode potential data measured for acidic 0.1 M $HClO_4$ aqueous electrolyte solutions at the Au(111) electrode with low concentrations of K_2SO_4 salt added.[7] Here, the most negative experimental surface charge density is about $\sigma_M \approx -(16-17)$ $\mu C\,cm^{-2}$ in qualitative accord with our computed most negative surface charge density. We note that the significant quantitative difference of our most negative computational bias potential and the corresponding experimental voltage is due to the idealized nature of our gold/water interfaces that do not contain electrolyte salts and, thus, cannot reproduce the experimental relation of electrode potential and surface charge density. Despite this limitation, inspection of the experimental data [7] suggests that our computed surface charge densities are qualitatively in the proper voltage regime before electrochemical reactions set in (which we clearly want to avoid given the declared purpose of the present study).

Supplementary Note 2 Collective variables to define the charge defects

Inspired by previous work [9], collective variables (CVs) are used to trace the positions of the H_3O^+ and OH^- charge defects using an in-house modified version of CP2K. The CV of the position of H_3O^+ or

OH^- is computed from

$$s = \frac{\sum_{i\in \mathrm{O}} z_i \exp(\lambda n_i)}{\sum_{i\in \mathrm{O}} \exp(\lambda n_i)}, \tag{2}$$

where λ is a large constant (with values of 20 and -20 for the acid and alkaline systems, respectively), O are the oxygen atoms of all water molecules in the sample and z_i are the positions of these O atoms on the z-axis. The number of hydrogen atoms n_i around an O atom i is computed from

$$n_i = \sum_{j\in \mathrm{H}} n^{\mathrm{H}}(r_{ij}), \tag{3}$$

where $n^{\mathrm{H}}(r)$ is a continuous function to count the number of H atoms around O atoms,

$$n^{\mathrm{H}}(r_{ij}) = \frac{1-(r_{ij}/r_c)^8}{1-(r_{ij}/r_c)^{16}}, \tag{4}$$

where r_{ij} is the distance between O and H atoms, and r_c is a cutoff radius (set to 1.27 and 1.11 Å for H_3O^+ and OH^-, respectively). This parameterization provides $n_i \approx 2$ for intact water molecules, ≈ 1 for OH^-, and ≈ 3 for H_3O^+ as required. Regardless of the exact values of n_i, this specific functional form of the CV will effectively filter out smoothly everything but the position of the oxygen with the most (H_3O^+) or least (OH^-) amount of hydrogen in the required bond distance range, thus yielding analytically differentiable ion positions along AIMD trajectories.[9]

We use these CVs to tag and to track the two charge defects (as depicted for instance in Fig. 1c in the main text) and to apply the mechanical wall potential in case of H_3O^+ to keep this defect close to the gold electrode at zero bias (see Supplementary Fig. 4).

Supplementary Note 3 Radial distribution functions in a slab geometry

Since solid/liquid interfaces are inhomogeneous and anisotropic in the z-direction versus the xy-plane, conventional spherical radial distribution functions (RDFs) cannot be used to quantify the particle distributions. Hence, we normalize the RDFs using a cylindrical volume instead following previous work [10–12]

$$g_{i,j}(r) = \frac{\rho_j(r)}{\rho} = \frac{n_{i,j}(r)}{2\pi r h \mathrm{d}r}, \tag{5}$$

where ρ is the density in the slab, $\rho_j(r)$ is the density of atom j at a distance r around atom i, $n(i,j)(r)$ is the number of j atoms at a distance between r and $r + \mathrm{d}r$, and h is the width of the slab.

Supplementary Note 4 Trajectories of H_3O^+

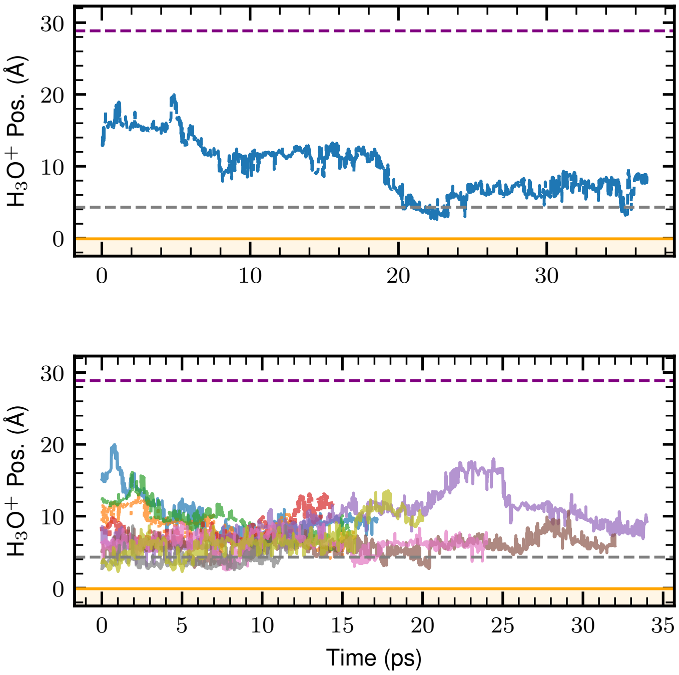

Supplementary Figure 3: H_3O^+ positions at finite surface charge density conditions corresponding to a computational bias of -3 V. The trajectory in the upper panel was monitored from the beginning of the charging stage. Additional trajectories in the lower panel were launched with new velocities of atoms after the trajectory in the upper panel reached a computational bias of about -3 V. Purple and gray horizontal dashed lines indicate the positions of the Ne electrode and the first water layer (the interface region IF, see main text), respectively, while the orange horizontal solid line represents the average position of the top Au layer.

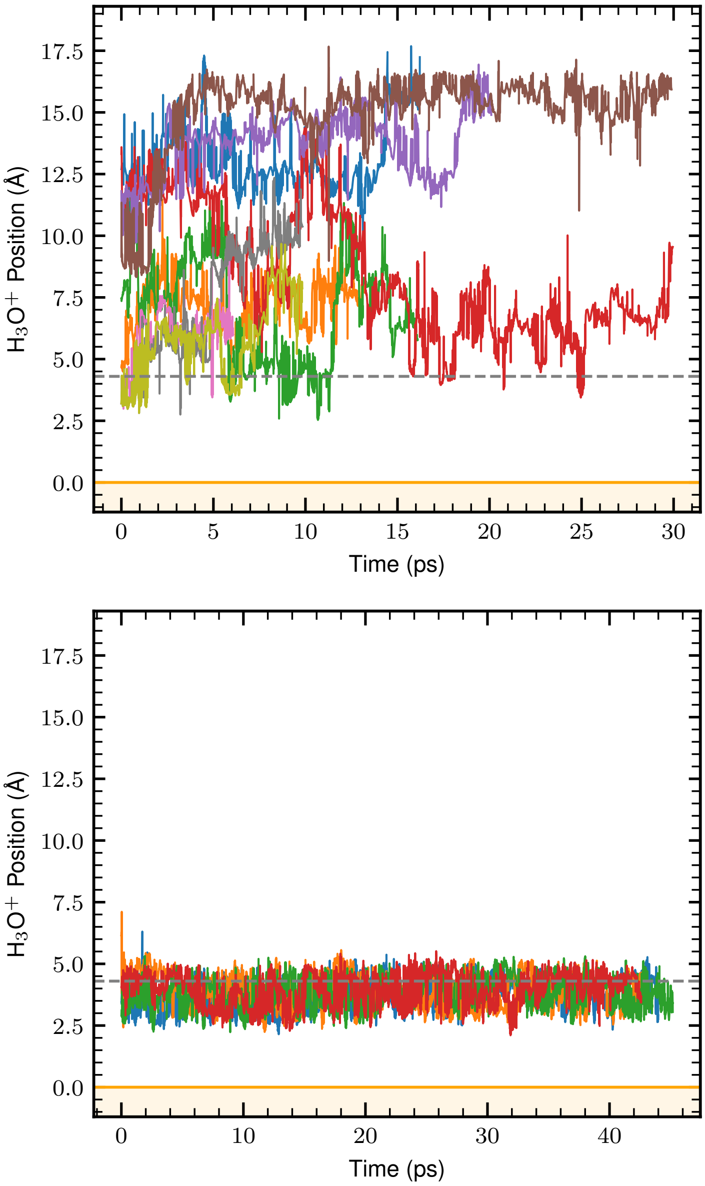

Supplementary Figure 4: Positions of H_3O^+ at zero surface charge density (pzc conditions) while applying a mechanical wall potential acting on H_3O^+ at 16.3 Å (upper panel) and 4.3 Å (lower panel), respectively, to force H_3O^+ to undergo Grotthuss-type charge migration in some proximity of the Au surface. Gray horizontal dashed lines and orange horizontal solid lines indicate the positions of the first water layer (the interface region IF, see main text), and the average position of the top Au layer, respectively. Note the different position scale compared to Supplementary Fig. 3.

Supplementary Note 5 Orientation and hydrogen bonding of water

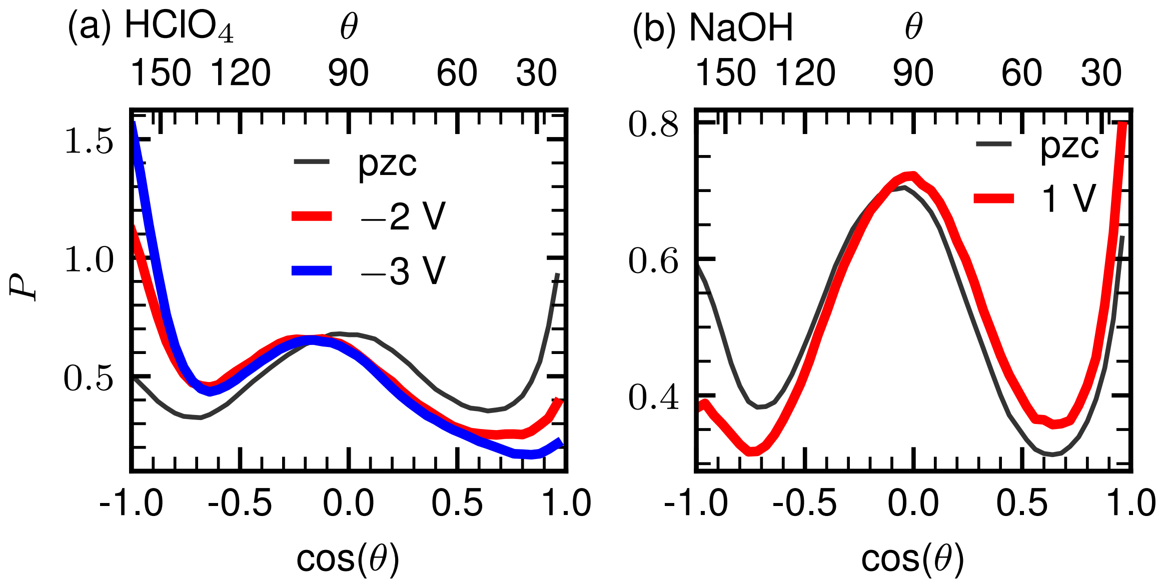

Supplementary Figure 5: Probability distributions of the cosine of the angle θ of OH vectors of water molecules in the (a) acidic ($HClO_4$ and (b) alkaline (NaOH) aqueous solutions at finite and zero surface charge density conditions corresponding to finite bias potential and pzc conditions as indicated; the surface charge densities corresponding to the reported bias potentials are compiled in Supplementary Table 1.

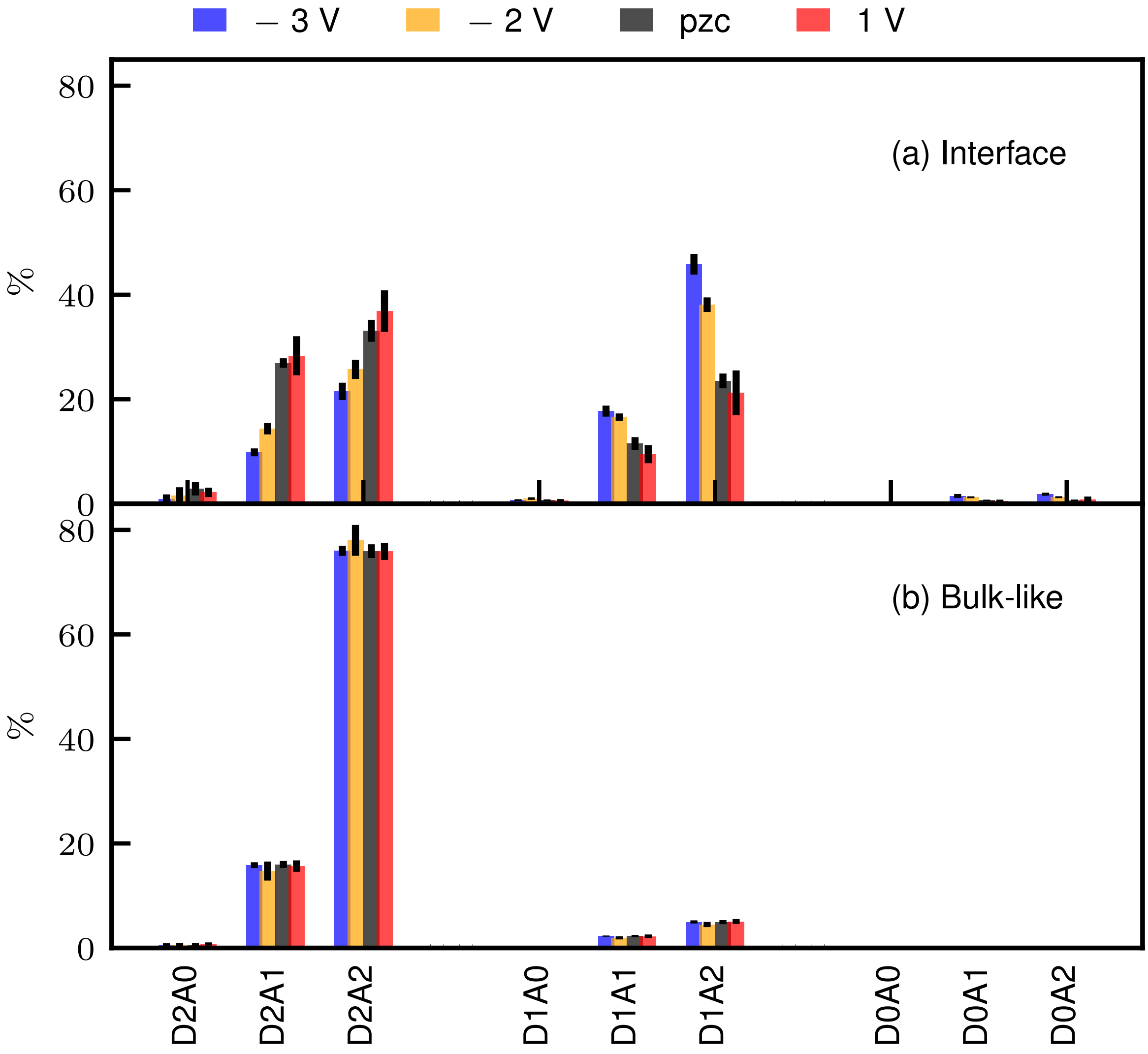

Supplementary Figure 6: Hydrogen bonding (HB) network statistics at finite and zero surface charge density conditions corresponding to finite bias potential and pzc conditions as indicated, where −3 V, −2 V and pzc relate to the acidic system ($HClO_4$) while +1 V to the alkaline system (NaOH); the surface charge densities corresponding to the reported bias potentials are compiled in Supplementary Table 1. The DnAm nomenclature that defined the HB patterns indicates that the corresponding water molecules are donating n and accepting m hydrogen bonds. Error bars indicate the standard deviation obtained by analyzing 11, 5, 13, and 12 trajectories for −3, −2, pzc, and +1 V, respectively.

Supplementary Note 6 Orientation and density profiles of H_3O^+

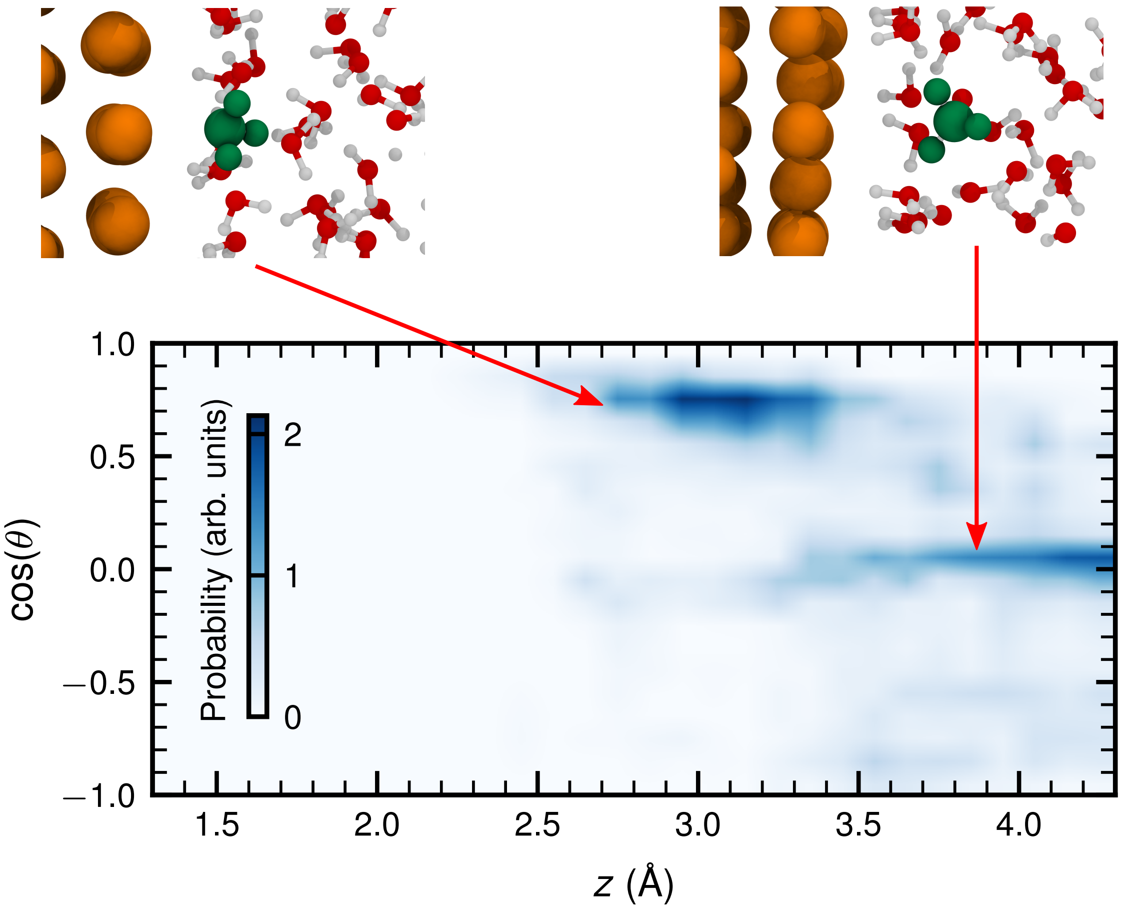

Supplementary Figure 7: Probability distribution functions of the cosine of the angle θ between the vector sum of the O−H bonds in H_3O^+ and the surface normal.

In case that the oxygen site of the H_3O^+ defect is rather close to the Au surface, around $z \approx 3$ Å according to Supplementary Fig. 7, its oxygen atom preferentially faces the Au surface, with its C_3 axis aligned to the surface normal, while its three hydrogen atoms form hydrogen bonds with neighboring water molecules, thus not pointing toward the electrode surface as illustrated in the representative top-left snapshot in Supplementary Fig. 7. This finding is in agreement with Vibrational Sum Frequency Generation (VSFG) spectroscopy concluding that the C_3 axis of H_3O^+ is aligned along the surface normal.[13] Alternatively, if one of the three H_3O^+ hydrogens oriented itself toward the electrode, the C_3 axis of H_3O^+ would be accordingly tilted and no longer perpendicular to the surface.

In contrast to this scenario, when H_3O^+ is located further away from the topmost gold layer (being located at $z = 0$ Å), the most probably orientation of the C_3 axis changes toward being perpendicular to the surface normal, and thus parallel to the gold surface, see Supplementary Fig. 7. As seen from the real-space configuration depicted in the top-right snapshot, H_3O^+ acts now as a bridge between water molecules at direct proximity to the Au surface and those that are further away. Note that the interface (IF) water layer extends up to roughly 4.3 Å. Interestingly, this orientation closely resembles the H_3O^+ configuration investigated by AFM experiments and DFT calculations,[14] where H_3O^+ is located slightly above a monolayer of water on the Au surface.

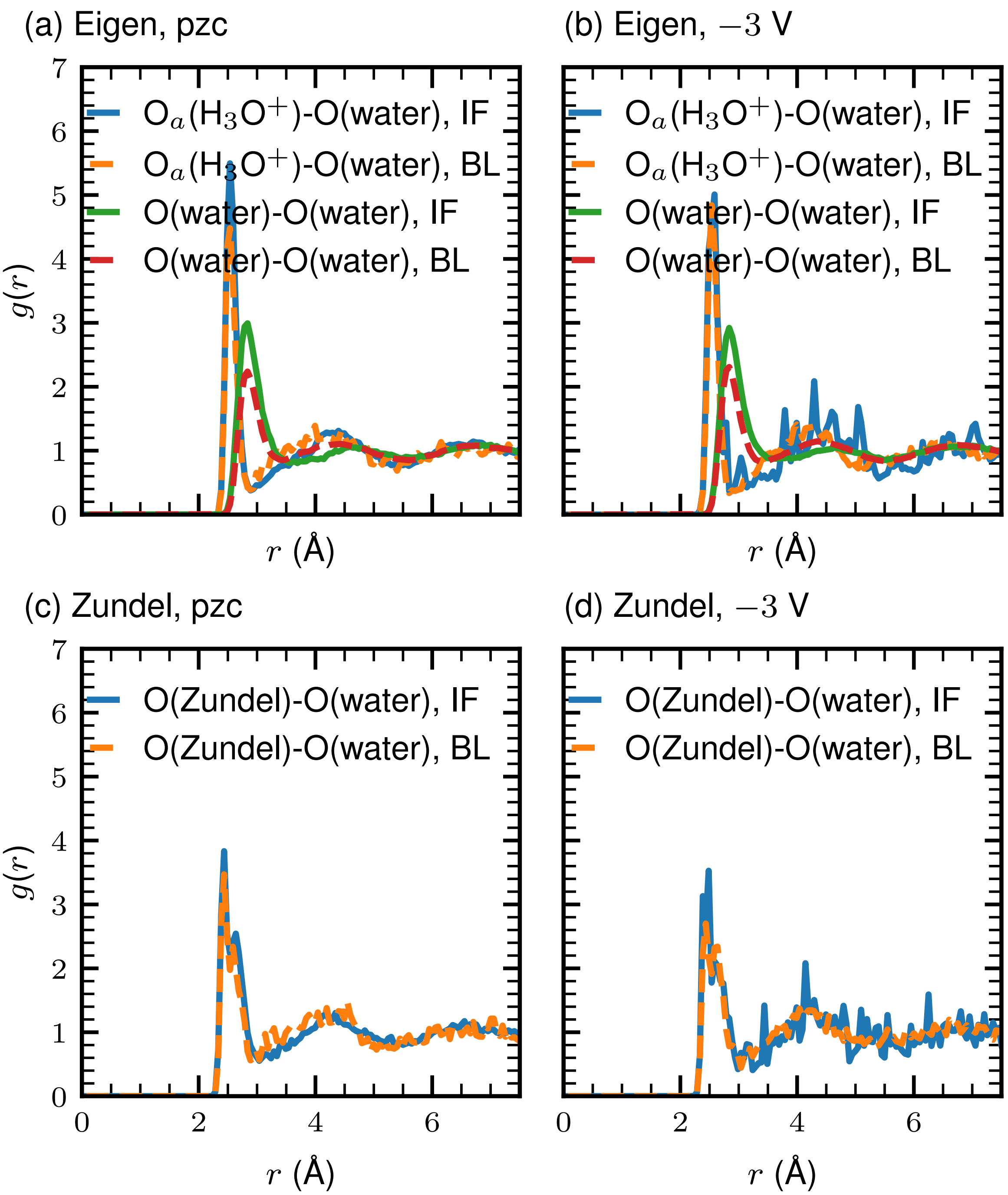

Supplementary Figure 8: Radial distribution functions of $O_a(H_3O^+)$/O(Zundel)-O(water) in (a, b) the Eigen and (c, d) Zundel states and O(water)-O(water) at the IF (interface) and BL (bulk-like) regions at finite and zero surface charge density conditions corresponding to finite bias potential and pzc conditions as indicated; the surface charge densities corresponding to the reported bias potentials are compiled in Supplementary Table 1. As the charge defects migrate via structural (Grotthuss) diffusion, the specific oxygen sites were identified for all AIMD configurations to ensure that the defect RDFs are consistently computed in the presence of charge migration.

Supplementary Note 7 Trajectories of OH^-

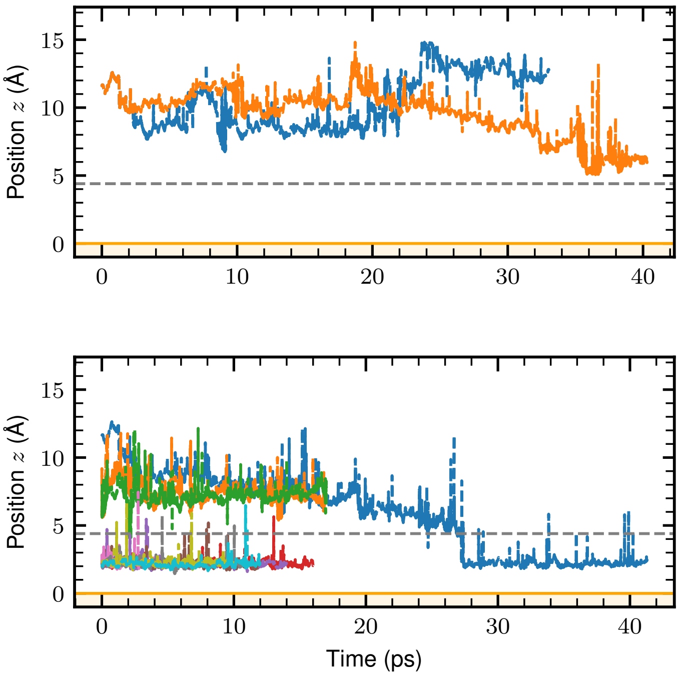

Supplementary Figure 9: Positions of OH^- at finite surface charge density corresponding to a finite bias of 1 V; the surface charge density corresponding to the reported bias potential is compiled in Supplementary Table 1. The trajectories in the upper panel were monitored from the beginning of the charging stage. Additional trajectories in the lower panel were launched with new velocities of atoms after the trajectories in the upper panel reached about 1 V. Gray horizontal dashed lines and orange horizontal solid lines indicate the positions of the first water layer (the interface region IF, see main text), and the average position of the top Au layer, respectively. Note the different position scale compared to Supplementary Fig. 3.

Supplementary Note 8 Density and excess charge profiles of H_3O^+ versus OH^-

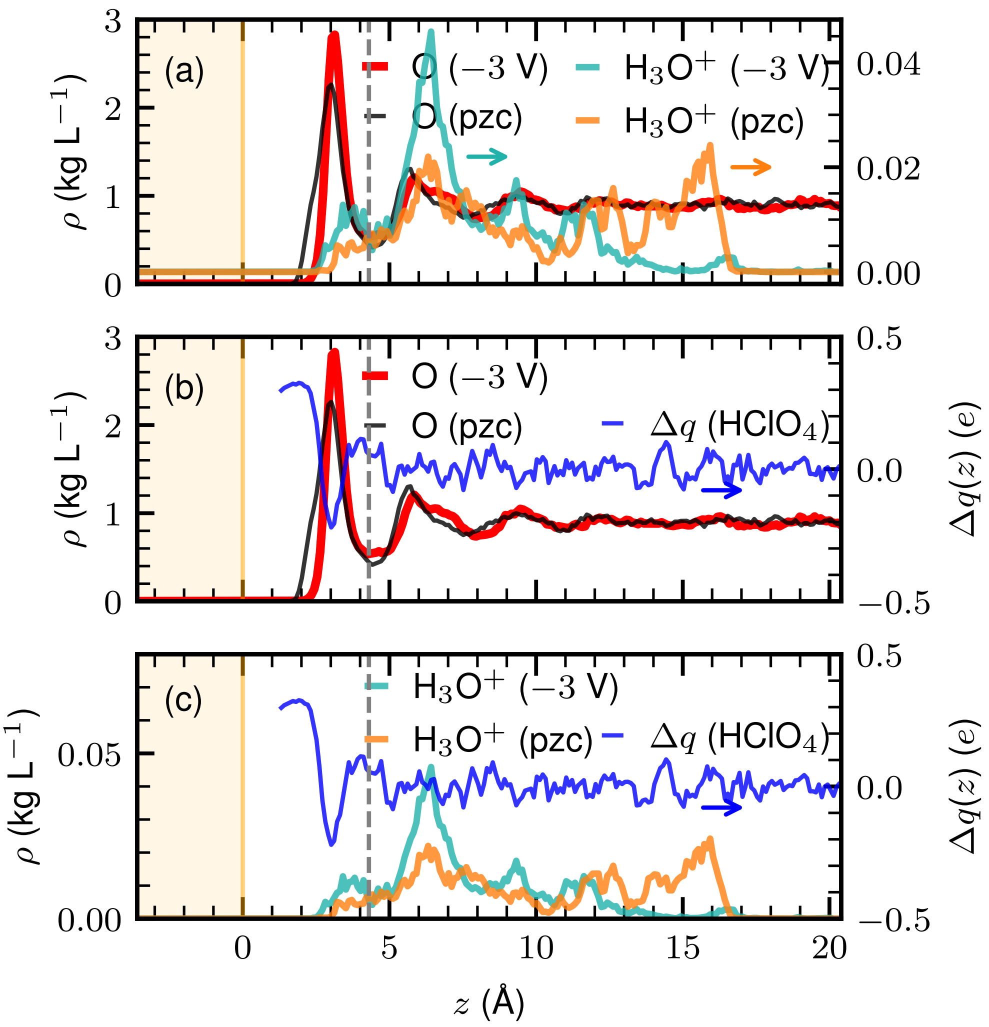

Supplementary Figure 10: (a) Density profiles of all O atoms as well as of the O sites of H_3O^+ (reported on right y-axis scale, see arrow) for the acidic solution at finite and zero surface charge density conditions corresponding to finite bias potential and pzc conditions as indicated; the surface charge densities corresponding to the reported bias potentials are compiled in Supplementary Table 1. (b) Density profile of all O atoms and the excess charge profile $\Delta q(z)$ (at −3 V, right y-axis) computed from the net atomic charge (NAC) of the oxygen and hydrogen atoms of the H_2O molecules along the surface normal. (c) Density profile of the O sites of H_3O^+ and the excess charge profile $\Delta q(z)$ (at −3 V, right y-axis).

Supplementary Figs. 10 and 11 present the density profiles of oxygen atoms in water molecules and in the two charge defects normal to the surface along z along with the corresponding excess charge profiles. The first figure indicates that H_3O^+ is able to approach the negative region of the excess charge around roughly 3 Å whereas its density profile in the IF region peaks at slightly larger distances where the excess charge starts to become positive. The free energy barrier for proton transfer near the surface suggests that the presence of the Au surface does not hinder proton transfer compared to the bulk phase (see main text, in particular Fig. 3d: compare IF to BL data). This behavior can be attributed to the flat solvation structure of H_3O^+, which can perfectly integrate itself into the existing two-dimensional hydrogen bond network near the surface via strong hydrogen bonding (given the essentially perpendicular arrangement of the C_3 axis of the hydronium ion). The resulting barrierless proton transfer process enables H_3O^+ to

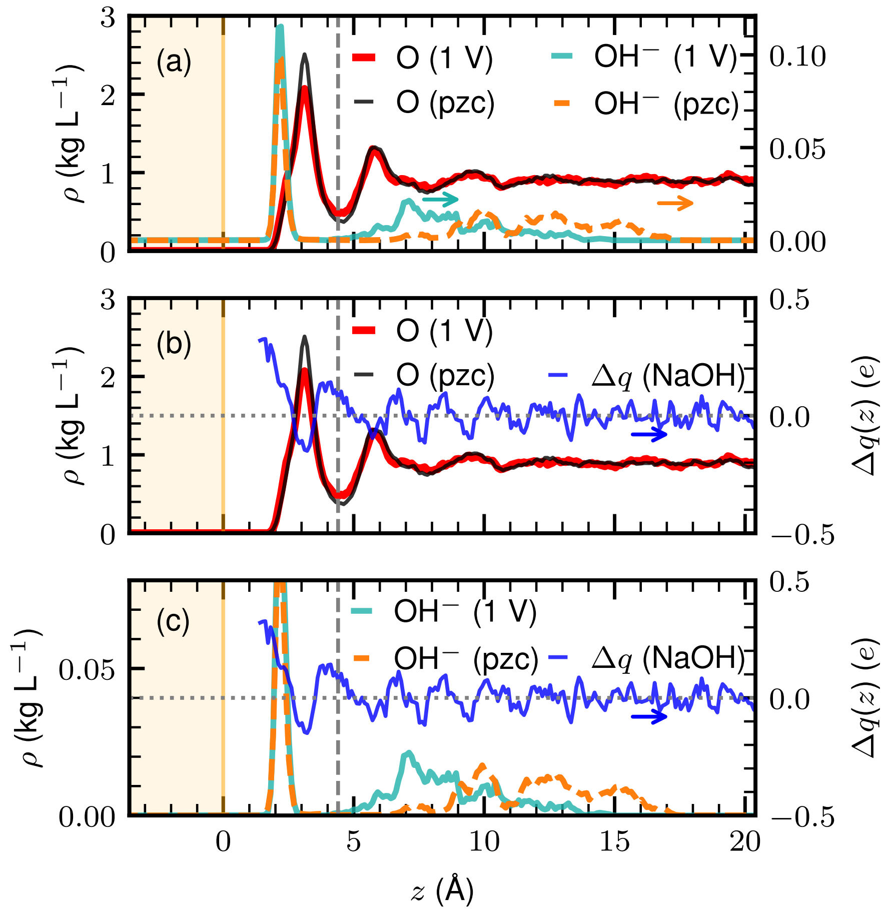

Supplementary Figure 11: (a) Density profiles of all O atoms as well as of the O sites of OH^- (reported on right y-axis scale, see arrow) for the alkaline solution at finite and zero surface charge density conditions corresponding to finite bias potential and pzc conditions as indicated; the surface charge densities corresponding to the reported bias potentials are compiled in Supplementary Table 1. (b) Density profile of all O atoms and the excess charge profile $\Delta q(z)$ (at 1 V, right y-axis) computed from the net atomic charge (NAC) of the oxygen and hydrogen atoms of the H_2O molecules along the surface normal. (c) Density profile of the O sites of OH^- and the excess charge profile $\Delta q(z)$ (at 1 V, right y-axis).

migrate in the region which corresponds to the first negative region of the NAC near the surface (around roughly 3 Å). However, at slightly larger distances, there is a change in orientation of H_3O^+ toward a more parallel arrangement of its C_3 axis w.r.t. the surface (see Supplementary Fig. 7) where the defect forms a bridge from the first to the second layer, being the region where the excess charge switches from negative to positive.

The OH^- ion is primarily localized around ≈ 2 Å which is deep in the interfacial (IF) region where the excess charge is positive, see Supplementary Fig. 11. We note that the excess charge is also positive around roughly 4 Å where no OH^- density is found, however there is simply not enough water at this distance that delimits the IF region that could host the defect. Once the water density is again sufficiently high beyond that first minimum, the propensity to find OH^- again correlates with the excess charge profile, for instance an increased population close to 7 Å is observed which is particularly pronounced at finite bias.

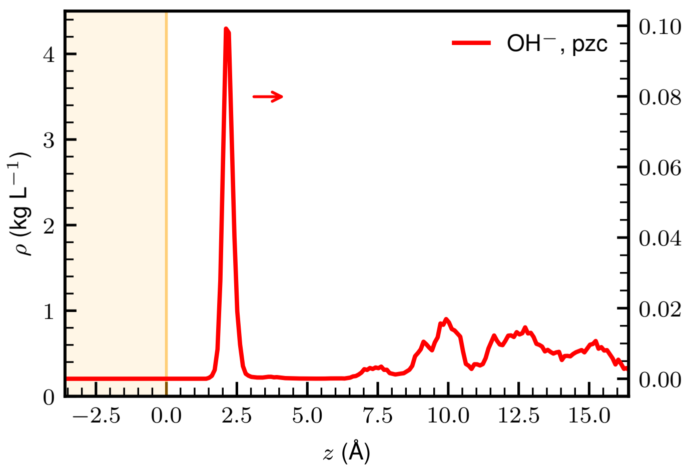

Supplementary Figure 12: Density profile of OH^- at zero surface charge density.

Supplementary Table 2: Donor and acceptor numbers of OH^- for the alkaline system (NaOH) at finite and zero surface charge density conditions corresponding to finite bias potential and pzc conditions as indicated; the surface charge densities corresponding to the reported bias potentials are compiled in Supplementary Table 1. The resting state is defined based on the charge transfer coordinate δ as $|\delta| > 0.5$ where $\delta = d_{\mathrm{O^*-H^*}} - d_{\tilde{\mathrm{O}}-\mathrm{H^*}}$ while the active state is defined as $|\delta| < 0.1$.

Voltage (V)	HB status	IF		IM		BL	
		Resting	Active	Resting	Active	Resting	Active
0	Donor	0.83	0.91	0.70	0.87	0.75	0.84
	Acceptor	2.74	2.60	4.41	3.58	4.43	3.80
1	Donor	0.85	0.93	0.85	0.83	0.82	0.82
	Acceptor	2.90	2.48	4.52	3.95	4.80	3.94

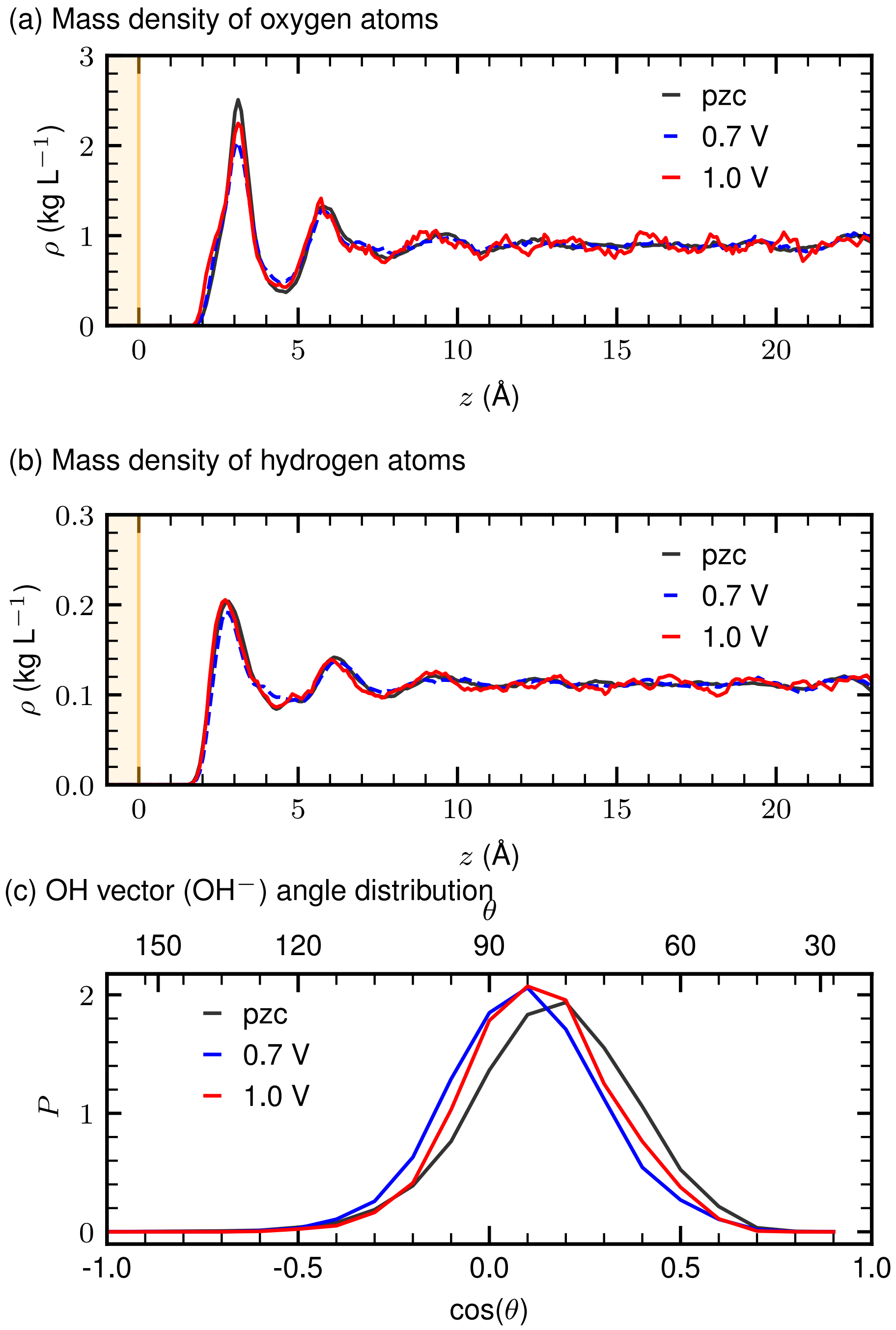

Supplementary Figure 13: Mass density profiles of (a) the oxygen and (b) hydrogen sites of the water molecules as well as (c) probability distribution functions of the cosine of the angle θ of the OH vector of OH^- with respect to the surface normal for the alkaline system at finite and zero surface charge density conditions corresponding to finite bias potential and pzc conditions as indicated; the surface charge densities corresponding to the reported bias potentials are compiled in Supplementary Table 1.

For refined analysis of the impact of finite surface charge density on the properties of the alkaline NaOH system, we split the set of underlying 1 V trajectories into 0.7 and 1.0 V as shown in Supplementary Fig. 13. The density profiles of oxygen atoms reveal noticeable changes already at a surface charge density corresponding to a computational bias potential of 0.7 V compared to the zero surface charge density (pzc) reference, and additionally the respective 0.7 V distribution function closely resembles that at a computational bias of 1 V, which yields the largest surface charge density that is supported by the OH^- system before reactions set in.

Supplementary Note 9 Net atomic charges

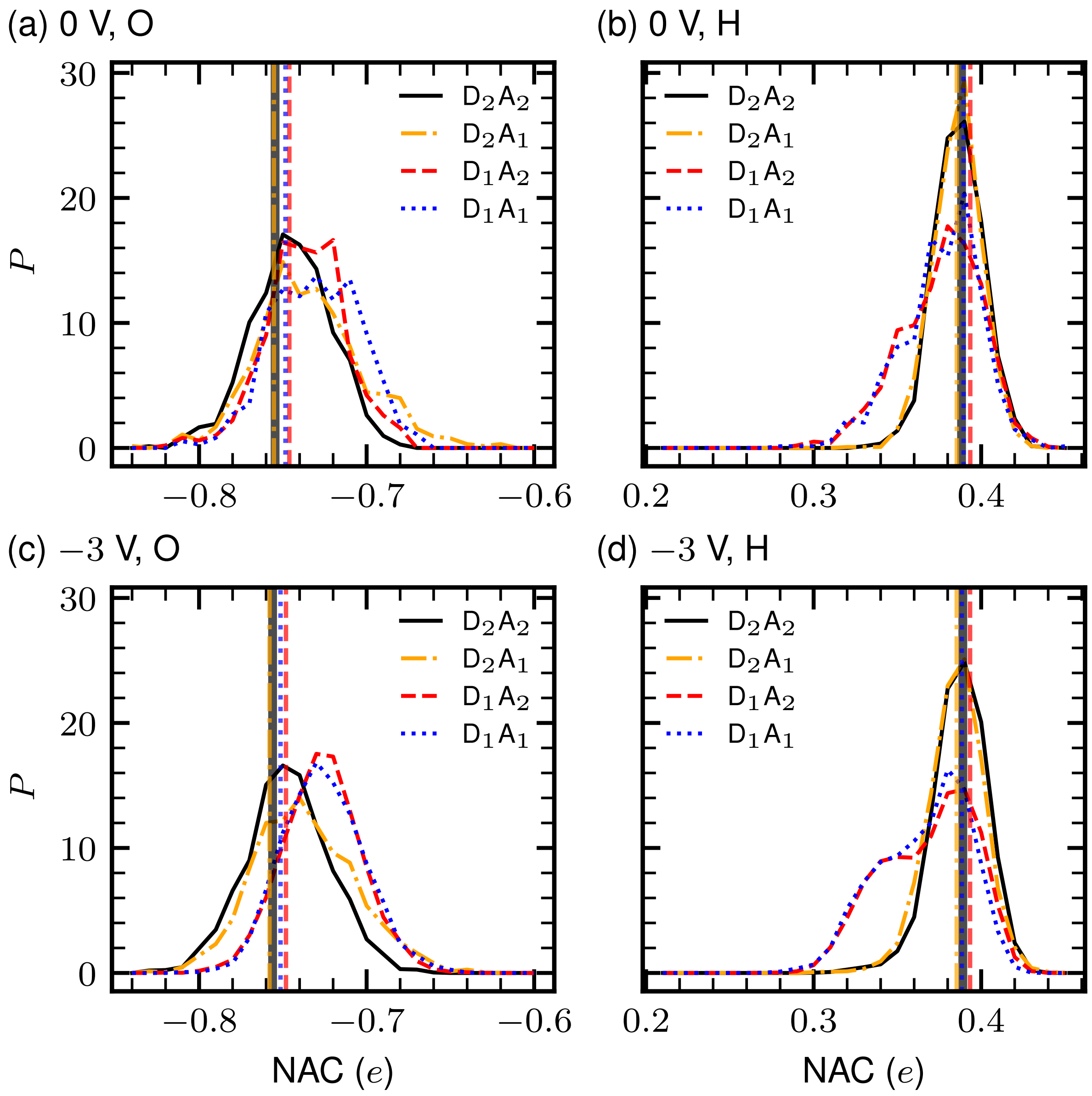

Supplementary Figure 14: Net atomic charges (NAC) of (a,c) oxygen (O) and (b,d) hydrogen (H) atoms in water molecules depending on their hydrogen bonding state in the $HClO_4$ system at finite (lower panels) and zero surface charge density (upper penels) conditions corresponding to finite bias potential and pzc conditions as indicated; the surface charge densities corresponding to the reported bias potentials are compiled in Supplementary Table 1. The vertical lines correspond to the values in the bulk-like (BL) region. The hydrogen bond patterns of water molecules are denoted as D_nA_m where n and m are the number of donor and acceptor bonds, respectively.

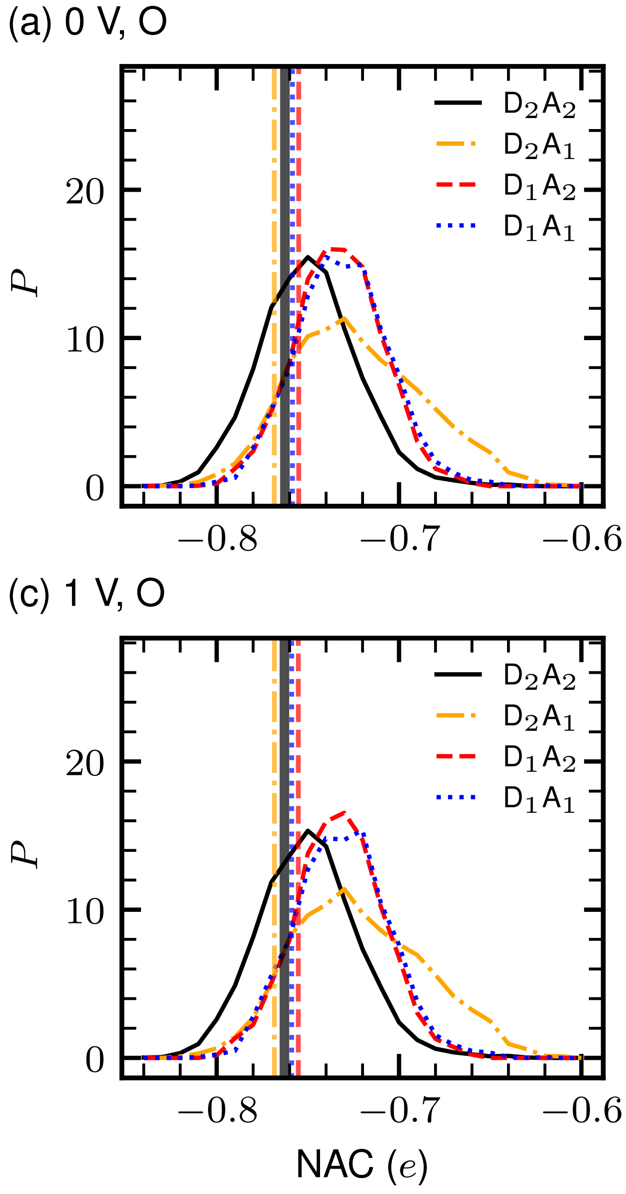

Supplementary Figure 15: Net atomic charges (NAC) of (a,c) oxygen (O) and (b,d) hydrogen (H) atoms in water molecules depending on their hydrogen bonding state in the NaOH system at finite (lower panels) and zero surface charge density (upper panels) conditions corresponding to finite bias potential and pzc conditions as indicated; the surface charge densities corresponding to the reported bias potentials are compiled in Supplementary Table 1. The vertical lines correspond to the values in the bulk-like (BL) region. The hydrogen bond patterns of water molecules are denoted as D_nA_m where n and m are the number of donor and acceptor bonds, respectively.

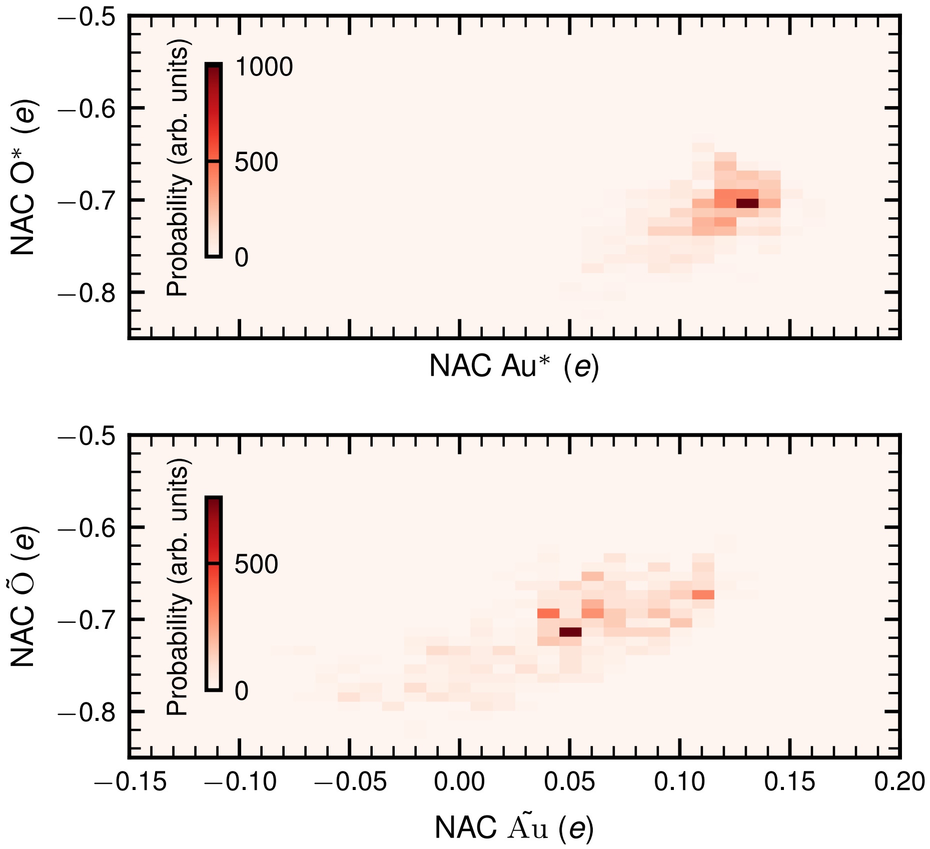

Supplementary Figure 16: NAC distributions of O^* as a function of Au^* (upper panel) and $\tilde{O}$ as a function of $\tilde{Au}$ (lower panel) at finite surface charge density corresponding to a finite bias of 1 V according to Supplementary Table 1; see main text for atom labeling.

Supplementary references

1. Marx, D. & Hutter, J. *Ab Initio Molecular Dynamics: Basic Theory and Advanced Methods* (Cambridge University Press, 2009).
2. Surendralal, S., Todorova, M., Finnis, M. W. & Neugebauer, J. First-principles approach to model electrochemical reactions: Understanding the fundamental mechanisms behind Mg corrosion. *Rev. Lett.* **120,** 246801 (2018).
3. Neugebauer, J. & Scheffler, M. Adsorbate-substrate and adsorbate-adsorbate interactions of Na and K adlayers on Al (111). *Phys. Rev. B* **46,** 16067 (1992).
4. Marx, D., Chandra, A. & Tuckerman, M. E. Aqueous basic solutions: hydroxide solvation, structural diffusion, and comparison to the hydrated proton. *Chem. Rev.* **110,** 2174–2216 (2010).
5. Le, J.-B., Fan, Q.-Y., Li, J.-Q. & Cheng, J. Molecular origin of negative component of Helmholtz capacitance at electrified Pt (111)/water interface. *Sci. Adv.* **6,** eabb1219 (2020).
6. Goldsmith, Z. K., Andrade, M. F. C. & Selloni, A. Effects of applied voltage on water at a gold electrode interface from ab initio molecular dynamics. *Chemical Science* **12,** 5865–5873 (2021).
7. Shi, Z., Lipkowski, J., Gamboa, M., Zelenay, P. & Wieckowski, A. Investigations of SO_4^{2-} adsorption at the Au (111) electrode by chronocoulometry and radiochemistry. *J. Electroanal. Chem.* **366,** 317–326 (1994).
8. Chen, A. & Lipkowski, J. Electrochemical and spectroscopic studies of hydroxide adsorption at the Au (111) electrode. *J. Phys. Chem. B* **103,** 682–691 (1999).
9. Park, J. M., Laio, A., Iannuzzi, M. & Parrinello, M. Dissociation mechanism of acetic acid in water. *J. Am. Chem. Soc.* **128,** 11318–11319 (2006).
10. Schoen, M., Diestler, D. & Cushman, J. Fluids in micropores. I. Structure of a simple classical fluid in a slit-pore. *J. Chem. Phys.* **87,** 5464–5476 (1987).
11. Das, B., Sharma, B. & Chandra, A. Effects of tert-butyl alcohol on water at the liquid–vapor interface: Structurally bulk-like but dynamically slow interfacial water. *J. Phys. Chem. C* **122,** 9374–9388 (2018).
12. Goujon, F., Malfreyt, P. & Tildesley, D. The pair distribution function in the planar gas–liquid interface: Application to the calculation of the surface tension. *J. Chem. Phys.* **151** (2019).
13. Tyrode, E., Sengupta, S. & Sthoer, A. Identifying Eigen-like hydrated protons at negatively charged interfaces. *Nat. Commun.* **11,** 1–7 (2020).
14. Tian, Y. et al. Visualizing Eigen/Zundel cations and their interconversion in monolayer water on metal surfaces. *Science* **377,** 315–319 (2022).
